# A Way to Boost the Impact of Business on 2030 United Nations Sustainable Development Goals: Co-creation With Non-profits for Social Innovation

**DOI:** 10.3389/fpsyg.2021.719907

**Published:** 2021-08-05

**Authors:** Yolanda Díaz-Perdomo, Luis Ignacio Álvarez-González, María José Sanzo-Pérez

**Affiliations:** Faculty of Economics and Business, University of Oviedo, Oviedo, Spain

**Keywords:** sustainable development goals, social innovation, value co-creation, business-non-profit partnerships, non-profit marketing

## Abstract

The evolution of Corporate Social Responsibility is forcing firms to adopt a new business approach based on combining competitiveness improvement with societal well-being. This evolution is materialized in the adoption of socially innovative practices to solve complex social problems, where collaboration is a key to confront them. And it is that, considering the existence of huge social and environmental challenges, independent actions undertaken by each of the societal actors with only their own resources reveal clearly insufficient to address them. Thus, a way firms can encourage the achievement of the Sustainable Development Goals (SDGs) is done by partnering with stakeholders, and particularly by developing the strategies of co-creation with non-profits. This study assesses the effects of business-non-profit value co-creation on both the organizational performance of the partners, and the social results linked to the SDGs. The methodology used to study the existence of these relationships is known as structural equations modeling (SEM) analysis. The results derived from a quantitative-based research with 205 Spanish non-profits show a positive effect of co-creation on indicators at the micro- (individuals), meso- (both the organizations), and macro-(society) levels. Furthermore, positive indicators at the micro- and macro-levels have a positive influence on the performance of the partners.

## Introduction

If we were to point out the main characteristics defining the present, which will certainly condition the future of human society, two phenomena would stand out in a special way. On the one hand, *interconnectivity* and *interdependence* as a result of the development of societies based on knowledge, digital transformation, and services, which generate an environment where boundaries between lucrative, public, and non-profit sectors are blurred. On the other hand, the existence of *huge social and environmental challenges*, covered by the 2030 United Nations Sustainable Development Goals (SDGs), that cannot be addressed from exclusively individual positions or through traditional welfare systems.

The crisis caused by the COVID-19 pandemic has only accelerated both trends (Chandra et al., 2021; Dube et al., 2021), showing the vulnerability of many apparently solid systems, and bringing out numerous initiatives by citizens based on a model of community that combines, at the same time, the local dimension with the globalization of knowledge. The production of sanitary materials in private homes or in companies dedicated to other type of products, the digital geolocation platforms that organize solidarity in nearby environments, or the development of open innovation projects to provide solutions to the pandemic are a few examples. A consequence of this reality is that innovation processes are open to the civil society. In fact, in the recent years, the term “social innovation” (SI) has been popularized to describe innovations that involve collaborative processes where the participation of citizens (from bottom to top) is significantly greater than in the traditional product innovations (Moulaert et al., 2017).

Thus, in the last decade, there have been worldwide institutional initiatives that drive SI, such as the Europe 2020 Strategy within the European Union, the World Economic Forum and its “Global Agenda Council on Social Innovation,” or the Local Employment and Economic Development (LEED) Forum on SIs of the Organization for Economic Co-operation and Development (OECD) (Harazin and Kósi, 2013; Morrar et al., 2017). From an academic point of view, SI has been addressed in a very fragmented and multidisciplinary way (Moulaert et al., 2005; Grimm et al., 2013; Van der Have and Rubalcaba, 2016). Such diversity reflects the fact that the term SI covers a wide range of actions developed not only by social entrepreneurs, non-profit organizations (NPOs), or the public sector, but also by companies.

The role of SI in companies has taken a parallel path to changes that have experienced the way of understanding the corporate social responsibility within them. This has evolved from a strictly philanthropic approach, through a more instrumental approach, where an economic return of these activities is already expected (for example, the implementation of cause-related marketing campaigns), until reaching an approach that combines the improvement of the competitiveness of the company with the improvement of the well-being of communities, by developing new business models and/or products/services that benefit the company and create value for society. In this regard, “enterprises are encouraged to adopt a long-term, strategic approach to CSR, and to explore the opportunities for developing innovative products, services and business models that contribute to societal wellbeing and lead to higher quality and more productive jobs” (European Commission., 2011, p. 8). This is what Pol and Ville (2009) already call “bifocal” innovations (profitable innovations that at the same time have a clear positive effect on the society).

An example that may illustrate this reality is a project launched in the mid-2000s by Coelce (subsidiary of the electricity company, Endesa Brasil) in Ceará, one of the poorest states in this country. The project aimed to address the environmental problem of waste, since in this state, there was a low awareness on the importance of recycling, and no infrastructures were available to carry out this process. In addition, Coelce had to face high levels of defaults by clients, thefts, and frauds. This initiative consisted of opening a new business line through the development of agreements with recycling companies and neighborhood associations, informal groups of garbage collectors, and other community institutions to organize a separate waste collection on certain specific points. Neighbors went to these places with their selected waste, which was weighted and valued. According to the assessment, neighborhood associations obtained discounts on the electricity bill, which were charged based on an identification card. Among other economic benefits achieved was the delinquency rate of the company dropping to 57%. From a social point of view, it was possible to reduce diseases like dengue fever, develop the recycling industry (creating direct and indirect jobs), and improve the recycling rate.

As can be seen in this case, a way through which companies can boost SI and contribute to achieve the SDGs involves the development of partnerships with NPOs, and more specifically, the development of co-creation projects with this kind of organizations. Acting as representative entities of civil society, the co-creation projects allow companies to get closer, and reinforce relationships with communities where they operate, promoting the participation of beneficiaries in innovative projects. Thus, greater interconnectivity of societies leads companies to interact with a wider range of target groups.

In conclusion, the main objective of the value of co-creation between firms and NPOs is to solve complex social problems (Voorberg et al., 2013). As a consequence, some of the stakeholders who previously occupied very peripheral positions become more strategic (Aarikka-Stenroos and Ritala, 2017), especially from the perspective of SI. This is the case of NPOs. In fact, a set of 17 SDGs explicitly includes as Goal 17 “partnerships for the goals,” highlighting that SDGs can only be realized with strong global partnerships and cooperation. However, in this context, “while partnerships between academia and other sectors are on the rise, there is a lack of relevant conceptual framework to practice and support impactful partnership development” (von Schnurbein, 2020).

The structure of the paper is as follows. Firstly, we present an approach to the concept of SI and its role in the achievement of SDGs. Following this, we reflect on how alliances between companies and NPOs can help enhance this type of innovation, focusing specifically on co-creation processes. Next, we reflect on the impact of co-creation processes in different estimates and results of both parties in the relationship and in the society as a whole. The description of the research carried out and its main results are included next. Lastly, the study details the main conclusion and the implications derived from the research.

## Conceptual Framework

### Social Innovation and SDGS

Social Innovation is a concept with many facets and implications. This has made disciplines and various approaches become interested in its analysis (Moulaert et al., 2005; Grimm et al., 2013; Van der Have and Rubalcaba, 2016). An overview of the different research traditions allows to highlight the existence of four main dimensions that share SIs (Howaldt and Schwarz, 2010; Grimm et al., 2013; Anheier et al., 2014; Van der Have and Rubalcaba, 2016): it is an innovation (either a product, a service, an organizational process, or any other type of activity), which is social in its *objectives* (it tries to address an important current social or environmental problem), in its *means* (innovation activities are developed through a collaborative process involving stakeholders), in its *long-term orientation* (with a focus on the sustainable use of resources and on future generations), as well as in its *final consequences and impact* (it pursues changes in practices and social behaviors until reaching a systemic change).

#### Innovations Aimed at Satisfying Social Needs

The first characteristic present in any SI is its orientation to social objectives. In other words, the focus is not on the purely personal or individual needs, but, on the concept of common good as the term, “social” implies (Howaldt and Schwarz, 2010). For example, (Mulgan, 2006, p. 146) conceives it as activities “motivated by the goal of meeting a social need and that are predominantly diffused through organizations whose primary purposes are social.” Likewise, for (Anheier et al., 2019, p. 17), “social innovations are seen as a solution for growing social, environmental and demographic challenges and as a result of the failure of conventional market capitalism, resource scarcity, climate change, ageing population and the associated care and health costs, globalization, and mass urbanization.”

A way of identifying what those objectives might be at the present time is implementing the 17 SDGs of the 2030 Agenda for Sustainable Development, adopted by the United Nations on September 25, 2015. In fact, after carrying out a systematic review and content analysis of the related literature, Eichler and Schwarz (2019) recently found that assigning 89% of the SI case studies to one or several SDGs clearly showed that SDGs are a suitable categorization system in the field of SI. In all these case studies, socially innovative practices are identified in different areas of activity that have substantial effects on the degree of compliance of SDGs.

#### Innovations Based on Collaborative Processes

Secondly, SIs are characterized by their collaborative nature, as reflected by sociological definitions described by Howaldt and Schwarz (2010, p. 65). Howaldt and Schwarz considered SI as “a new configuration of social practices in certain areas of action or social contexts prompted by certain actors or constellation of actors in an international, targeted manner with the goal of better satisfying or answering needs and problems than is possible on the basis of established practices.”

This characteristic is the second dimension suggested by Moulaert et al. (2005, p. 1976), who understood SI as “[c]hanges in social relations, especially with regard to governance, that enable the above satisfaction, but also increase the level of participation of all but especially deprived groups in society.” Under this perspective, SI is “good for society and enhance society's capacity to act” (Murray et al., 2010, p. 3), and “social innovation can refer both to the means and the ends of action” (Grimm et al., 2013, p. 438). This dimension reflects that SI practices are “a higher degree of bottom-up and grass-roots involvement than technological innovation” (Anheier et al., 2019, p. 19), so that “the process is part of the outcome and social innovation is an end in itself” (Grimm et al., 2013, p. 438).

The different research streams have highlighted different forms of developing these collaborative processes. On the one hand, research on local development has explored news ways of governance and participation in neighborhoods and regions (Moulaert et al., 2005). One example for this is the social platforms of collective action. On the other hand, studies on open innovation (Chesbrough, 2003; Zhu et al., 2019) have used a network analysis to show how companies (and individual themselves) can collaborate with individuals and communities for the exchange of knowledge. An example can be found in the open virtual communities that have worked to mitigate health, social, economic, and environmental impacts of the pandemic caused by COVID-19. Meanwhile, in service marketing literature, the approach based on the service dominant logic (Vargo and Lusch, 2004) has highlighted the possibilities of co-creation processes in the design and/or service provision.

#### Sustainable Innovations

Business and economic literature, as well as work focused on entrepreneurship and innovation, have emphasized factors like “improvement” or “creativity,” from a classic definition of innovation as a process of creative destruction. Following this approach, (Phills et al., 2008, p. 36) postulated that SI is a “novel solution to a social problem that is more effective, efficient, sustainable, or just than existing solutions and for which the value created accrues primarily to society as a whole rather than private individuals.”

This dimension cannot only be interpreted in terms of innovation. Innovation needs to incorporate the notion of long-term sustainability. Only if the innovation tries to satisfy a current social need without compromising the capacity of future generations to satisfy its own needs, it can be regarded as truly social.

#### Innovations Generating a Change in Social Behaviors

Any attempt to describe an SI should include a range of qualifiers that measure the extent to which this activity has changed certain behaviors or social practices, focusing on the change that arises as a result of the activities. In addition, as stated by Howaldt and Schwarz (2010, p. 32), the spread of an SI can be understood as a “process through which the social ideas and inventions spread through existing communication paths in a social system.” Therefore, the acceptance of an SI cannot be seen as limited to a social agent acting alone, but always linked to social environments. This explains why literature usually highlights three levels of analysis in terms of behavioral changes generated by SI: micro, meso, and macro levels.

Results at the micro level involve a change in behaviors developed by direct beneficiaries or other individual stakeholders. This level also incorporates the degree of empowerment achieved. Results of meso (organizations) include changes in organizational behaviors like improving the collaborative nature of the government system in the organization or the introduction of good governance practices. Lastly, the macro level (society) is connected to long-term changes and systemic changes. Concerning this subject, (Dentoni et al., 2018, p. 334) highlighted two dimensions of the systemic change: breadth, that implies an interconnected change in multiple areas and subsectors of activity, and depth, that “entails a power shift among actors in society.”

### Business-Non-profit Value Co-creation as a Driver of Social Innovation

Partnerships among public, business, and non-profit sectors have represented a trend that has been mentioned for decades (Austin and Seitanidi, 2012a; Clarke and Crane, 2018). The typology includes many different alternatives, covering the corporate philanthropy, corporate foundations, license agreements, sponsorships, cause-related marketing campaigns, the development of joint awareness and communication campaigns, corporate volunteering programs, pro-bono activities, or even joint ventures or hybrid organizations for the development of new products or services.

These possibilities differ in the type of value generated, the associated costs and risks, and their governance structures, being usual to distinguish a “continuum of collaboration,” where several forms of collaboration are identified (Austin and Seitanidi, 2012a), ranging from “philanthropic collaborations” —characterized by unilateral directionality of the resource flow (basically cash), from the company to the non-profit—to transformational partnerships, in which both parties agree “on their intention to deliver transformation through social innovation bettering the lives of those afflicted” (Austin and Seitanidi, 2012a, p. 743).

The capacity of partnerships at advanced stages of the collaboration to promote SIs within the company stems from the legitimizing role that NPOs can play among their beneficiaries and the civil society they represent. Thus, a recent study by (Weerawardena et al., 2021, p. 763) acknowledges that “in pursuing their dual value focus, SPOs [social purpose organizations] need to engage with a broad and diverse set of internal and external stakeholders [] which means they have to balance shifting ‘institutional logics' []. The challenges thus lay in reconciling active stakeholders focused on advancing particular logics and who are situated at the endpoints of the social-economic value creation spectrum. In doing so, SPOs have to provide criteria for legitimacy and gain acceptance in the eyes of stakeholders.” In the same sense, (Le Ber and Branzei, 2010, p. 603) postulated that “beneficiaries often remain marginalized during value creation processes, and thus many of their potential contributions may fail to materialize.”

Therefore, NPOs can reinforce the role of the beneficiaries and strengthen the legitimacy of the company with which they strategically cooperate (Anheier et al., 2014; Cajaiba-Santana, 2014). In fact, the four dimensions that make up an SI would be enhanced. Firstly, since the essence of NPOs is addressing social needs, their strategic collaboration with the company can be useful to incorporate the social dimension specified in the SDGs in the economic objectives of the latter. Secondly, NPOs usually keep direct and close channels of communication with beneficiaries, since they are governed and managed by their main stakeholders, and their capacity to mobilize beneficiaries and/or volunteers can be significant (Anheier et al., 2014). Therefore, if a company participates in a partnership in which the level of interaction is more intense, the NPO is also more likely to be willing to promote the participation of their beneficiaries in the company activities. Thirdly, the collaboration with NPO can contribute to moderate the excessive orientation to short-term results that companies can face as a consequence of the competitive pressures to which they are subjected. Fourthly, the systemic change requires multiple interconnected changes that spread through individuals, institutions, regulations, organizations, and activity sectors, which means that intersectoral partnerships may be essential to achieve it.

One of the specific aspects characterizing transformational partnerships is the existence of value co-creation (Austin and Seitanidi, 2012a). Research on value co-creation has significantly proliferated in the recent years (Grönroos, 2012; Bharti et al., 2015; Ranjan and Read, 2016; Vargo and Lusch, 2016). According to (Grönroos, 2012, p. 1520), value co-creation can be defined as “the joint actions by a customer (or another beneficiary) and a service provider during their direct interactions.” After conducting a thematic content analysis of value co-creation literature, Bharti et al. (2015) have identified 27 elements related to co-creation, which are classified into five critical dimensions: process environment, resources, co-production, perceived benefits, and management structure.

The so-called, “co-production” includes the central characteristics of co-creation (Bharti et al., 2015): (1) customer participation: the extent to which the client, or other type of stakeholder, provides suggestions, shares information, and collaborates in decision-making processes with the company; (2) customer involvement: the participative and dynamic connection (learning) of clients with the company; (3) partnership and engagement: promoting the meaningful physical, cognitive, and emotional participation of the company and its employees with customers, and the long-term relationship between both partners; and (4) mutuality: proactivity and receptivity toward the other party in the relationship based on mutual interest, in other words, openness toward the influence of the other party, to the extent that there is availability and predisposition to change depending on the circumstances of the partner.

This management approach provides an understanding of how companies, customers, and other market participants co-create value through their interactions (Vargo and Lusch, 2004). Thus, the co-creation of value aims to serve the interests of all stakeholders who are part of the value chain of the company (Ramaswamy, 2009). In this context, co-creation research has recognized that “value co-creation must be understood in the context of relationships among a complex web of actors (customers, employees, suppliers and other stakeholders)” (Vargo and Lusch, 2010, p. 177). In this way, co-creation activities can be extended to a diverse set of potential partners, including NPOs (Ramaswamy, 2009).

In this respect, NPOs are assigned a particular potential to promote value co-creation in the SI context, as entities that “engage in advocacy activities to protect or advance the position in society and welfare of people needing help, e.g., disabled or poor persons, or members of neglected communities” (Anheier et al., 2019, p. 3). Therefore, the value co-creation between both sectors, and the coordinated action between companies and NPOs through the implementation of intersectoral partnerships, can improve the company and the NPO processes, and externally extend such transformation through SI to solve complex problems the society faces (Sanzo et al., 2015).

With those arguments, it is important to analyze, from the NPO perspective, to what extent the adoption of the value co-creation strategy between companies and NPO can increase the social action of the company on the NPO. For this purpose, it can be assumed that the value co-creation strategy that NPOs develop in their collaborative relationships with companies would imply: (1) the participation of the company in the different stages of the collaboration process (participation); (2) that each partner gives and receives in the same proportion as the other party in the relationship (reciprocity); (3) the promotion of a dynamic learning process through the acquisition of knowledge (learning); and (4) the existence of effective participation between the two parties to foster a long-term relationship between the company and the NPO (engagement).

### Consequences of Business-Non-profit Value Co-creation

Three kinds of different indicators stand out for measuring the social performance, i.e., outputs, outcomes, and impacts. According to Lee and Nowell (2015), (1) outputs involve the physical products obtained or the activities implemented (in our case, as the result of the collaboration process); (2) outcomes refer to the effect of products or activities conducted on the behavior or environmental conditions through the services performed (the difference made by outputs); and (3) the measurement of social impacts focuses on the contribution and benefits oriented to the community and to the society as a whole during a certain period of time.

An example that shows the difference between outputs, outcomes, and impacts can be seen through a program aimed at spreading the use of a certain medicine: outputs can be measured by the amount of medicine provided by the program (or by the number of possible training courses implemented or the number of people who have attended these courses); outcomes represent the use of medications by patients or the changes in their behaviors regarding this medicine, whereas the impact reflects the ultimate effects on the health of the patients from the use of the medicine, compared to a situation in which they would not have taken it (Van Tulder et al., 2016).

Traditionally, the most common types of measurement indicators employed to assess social performance have been the outputs (LeRoux and Wright, 2010). However, outcomes and impacts have replaced the use of inputs (e.g., the income of an organization) and output measurement indicators (e.g., the number of programs implemented and/or of beneficiaries who attended) (MacIndoe and Barman, 2013; Ebrahim and Rangan, 2014), since inputs and outputs do not reflect the real changes generated in social practices as a result of the program, but only the resources employed and/or the activities implemented leading to these changes.

In addition, social performance measures may not offer a complete and in-depth view of the real impact of the project or the organization if an appropriate level of analysis is not taken into account (Van Tulder et al., 2016). Literature on this topic allows us to identify three basic levels of analysis when assessing the consequences of the collaboration between companies and NPOs (Kolk, 2013): (1) micro (benefits for individuals), (2) meso (organizational benefits), and (3) macro (impact on society). Thus, when the collaboration moves from sole creation to value co-creation, greater value, and outcomes are expected at the three levels (Austin and Seitanidi, 2012b).

**Figure 1:**
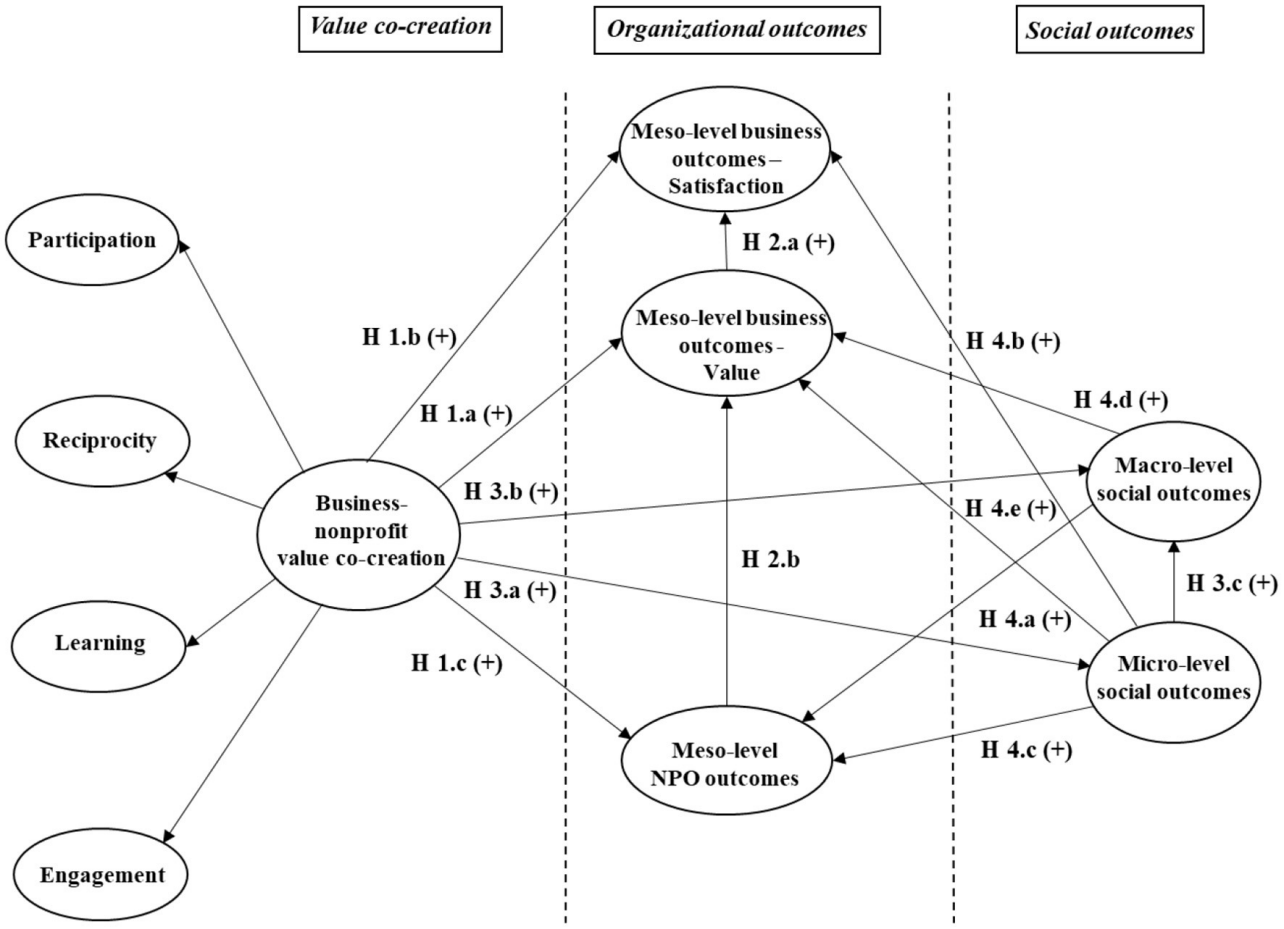
Consequences of the business–NPO value co-creation.

Outcomes of co-creation are usually analyzed at the meso level, as “the most common focus in the literature and in practice is on the value accruing to the partners, which are the organizational benefits that enhance the performance of the non-profit or the company” (Austin and Seitanidi, 2012b, p. 945). However, the analysis of performance indicators at the micro and macro levels is also essential to understand the consequences of value co-creation, given that the use of these indicators is vital to evaluate the improvement of programs implemented to solve the economic, social, and environmental problems the society currently faces (Zainudin et al., 2020). Consequently, it seems necessary to establish potential consequences of value co-creation both on organizational and social outcomes and the possible interlink between these two levels of consequences (Figure 1).

#### Consequences of Business-NPO Value Co-creation on Meso-Level Outcomes

Outcomes at the meso level are the benefits or changes in behavior that occur in organizations that implement the collaboration (the company and the NPO) (Van Tulder et al., 2016). Overall, different types of value can be derived from such a type of partnership for both partners (Austin and Seitanidi, 2012b): associational value (improved visibility and credibility), transferred value (cash, in-kind gifts, etc.,), interaction value (opportunities for learning and access to networks), and synergistic value (innovation).

In the case of the business partner, the development of social projects co-created with an NPO could help the company obtain relevant knowledge from the developed joint activity and access to new targets (Yaziji and Doh, 2009), thus providing more valuable products and/or services to its customers—if it acquires information from the NPO that can be disseminated and applied within its organization—and making changes in its management as a result of the collaboration. This learning, together with a solid and effective engagement in the development of collaborative social projects, could also improve the image and/or competitive position of the company in the society (Spitz et al., 2021). Thus, the following hypotheses are derived:

**Hypothesis 1.a**. Business-non-profit value co-creation influences positively on the meso-level value business outcomes.

Likewise, co-creation aims to serve the interests (improve their satisfaction, acquire better skills.) of all parties involved, and focuses on their experiences and how they interact with each other (Ramaswamy and Gouillart, 2010). In fact, the decision to adopt co-creation practices leads to meeting expectations, maintaining effective communication, and ensuring the satisfaction of the stakeholders involved in the process (Payne et al., 2008). Therefore, the satisfaction of the company with the result and the impact of the partnership could increase if co-creation processes are carried out with the NPO. In consequence,

**Hypothesis 1.b**. Business-non-profit value co-creation influences positively on the meso-level satisfaction business outcomes.

On the other hand, the NPO could improve its outcomes as a result of establishing partnerships with companies (Al-Tabbaa et al., 2021). In the context of business-NPO co-creation (Bharti et al., 2015), the improvement of the outcomes of the NPO would occur as a consequence of: the participation of the company in each stage in the collaboration process, the reciprocity between both partners, the presence of learning processes, and the effective engagement of the company with the NPO. Thus, increasing visibility, reputation, and/or legitimacy of the NPO, or improving the efficiency and productivity of its staff, would be changes made in the organization as a result of the collaboration with the company. Therefore,

**Hypothesis 1.c**. Business-non-profit value co-creation influences positively on the meso-level NPO outcomes.

#### Meso-Level Outcomes Influences

Co-creation focuses on the importance of the active participation of all stakeholders for their effective interaction (Ranjan and Read, 2016). In fact, the participation of the partners in the co-creation allows for the company to satisfy personalized demands and achieve competitive advantages (Zhang and Chen, 2008), that provide benefits to the company in terms of satisfaction (Bharti et al., 2015). Thus, co-creation with NPOs improves the image of companies (Kolk et al., 2010), which could influence them to be more satisfied with the results of the collaboration. Thus,

**Hypothesis 2.a**. Meso-level value business outcomes influence positively on the meso-level satisfaction business outcomes.

Likewise, the benefits obtained by each of the partners in the partnership derived from the resources provided by the other party to the relationship (for example, a new skill learned from a partner) (Austin and Seitanidi, 2012a, p. 731). Furthermore, according to Yaziji and Doh (2009) “working with businesses to improve a particular issue can be a way of showing that the non-profit organization can influence business practices” (Spitz et al., 2021, p. 4). Thus, the fact that the NPO obtains better results, especially in terms of visibility and reputation, can also reinforce the positioning of the company as a responsible organization in the eyes of society, increasing its performance indicators. Therefore,

**Hypothesis 2.b**. Meso-level NPO outcomes influence positively on meso-level value business outcomes.

#### Consequences of Value Co-creation on Social Outcomes

Social outcomes are mainly linked to the analysis of performance indicators at the micro and macro levels, and their measurement is essential to analyze the implementation and results of the SDGs (Zainudin et al., 2020).

The micro level involves measuring or analyzing the consequences of the business–NPO collaboration on the individuals involved in the alliance, such as the beneficiaries or users of the organization (Van Tulder et al., 2016). The outcomes at the micro level are the benefits or changes in these people as a result of the NPO–business collaboration (Van Tulder et al., 2016), such as the satisfaction of the beneficiaries or the needs of the users (in case of having attended a course on employability, an outcome could be the number of people who have obtained a job). Thus, the business–NPO collaboration, through the combination of their respective resources and capacities, can produce benefits for the individuals (beneficiaries) targeted by the organizations that carry out the projects, activities, etc. (Austin and Seitanidi, 2012b). Consequently, we expect that:

**Hypothesis 3.a**. Business-non-profit value co-creation influences positively on micro-level indicators of social outcomes.

For its part, business–NPO collaborations can produce social, environmental, and economic values for society as a whole (Austin and Seitanidi, 2012b). Ebrahim and Rangan (2014, p. 118) defined social impact as “lasting changes in the lives of people and their societies.” These changes can entail positive or negative effects, mainly in the long term, produced by the alliance directly or indirectly, intentionally, or involuntarily (OECD, 2002). For example, the creation of opportunities for sustainable economic growth in a certain community or the improvement of the sectoral conditions of the social environment in which the collaboration under evaluation is developed would be clear examples of social impacts generated at the macro level as a consequence of the co-creation processes between the company and the NPO. It is difficult that a single organization, working only with its own resources, can lead to these long-term changes. In this sense, “firms seem keen to embrace strategic collaboration opportunities that would result in lasting projects to augment the overall impact of the initial business investment, since the project should eventually become self-sustaining and then deliver ongoing value to society and the NPO” (Al-Tabbaa et al., 2021, p. 21). Therefore, an organization fostering partnerships can produce value co-creation and social impact as the alleviation of economic inequalities (Zhu and Sun, 2020). Consequently, we propose:

**Hypothesis 3.b**. Business-non-profit value co-creation influences positively on macro-level indicators of social outcomes.

Finally, “these two levels of sustainability are interlinked. Micro-level sustainability affects macro-level sustainability through small actions of individuals and organizations or businesses around the world” (Zainudin et al., 2020, p. 10). In fact, Rousseau (1985) and House et al. (1995) would emphasize the need to develop, on collaboration, a “meso” paradigm, that is, the simultaneous study of at least two levels of analysis (micro and macro), and their reciprocal relationships, indicating the need for this perspective and some basic concepts and principles of multilevel research (Molina-Azorín et al., 2020, p. 323). Thus, benefits obtained by the users of the services, activities, projects, or programs developed jointly by the company and the NPO could lead to the achievement of a positive social impact on the community where those activities and services are developed as a result of collaboration between both partners. So,

**Hypothesis 3.c**. The micro-level indicators of social outcomes influence positively on macro-level indicators of social outcomes.

#### Consequences of the Social Outcomes of Value Co-creation on the Organizational Benefits of the Partners

At the meso level, “the groups of actors are connected by dyadic partnerships. These factions provide a connection between micro level partnership dyad and the macro or the whole structure of the partnership field” (AbouAssi et al., 2021, p. 4). Thus, it is important to note that the benefits at the micro level “may spill over to the organizational level (meso level), impacting both the company (e.g., in terms of an improved corporate image), and the non-profit partner (e.g., in terms of more visibility or access to more resources)” (Kolk et al., 2010, p. 135). For example, the NPO can attain better results by an increase in the number of people who wish to collaborate as donors or volunteers; the company can improve the sales of the products designed for those targets. In consequence, the following hypotheses are derived:

**Hypothesis 4.a**. The micro-level indicators of social outcomes influence positively on the meso-level value of business outcomes.
**Hypothesis 4.b**. The micro-level indicators of social outcomes influence positively on the meso-level satisfaction of business outcomes.
**Hypothesis 4.c**. The micro-level indicators of social outcomes influence positively on the meso-level of NPO outcomes.

Furthermore, “outcomes at the meso level are likely to be positively related to those at the macro level” (Kolk et al., 2010, p. 135). Thus, NPOs appear stronger when entering into partnerships and hence create more value, not only to support their beneficiaries but also to enhance their long-term sustainability (Al-Tabbaa et al., 2021, p. 26), achieving a positive social impact in the community where they operate (Austin and Seitanidi, 2012a), and improving partner performance (AbouAssi et al., 2021). In this sense, for instance, the creation of opportunities for sustainable economic growth in the community could reinforce the competitive position of the company or the fulfillment of the NPO mission. Consequently, we expect that:

**Hypothesis 4.d**. The macro-level indicators of social outcomes influence positively on the meso-level value of business outcomes.
**Hypothesis 4.e**. The macro-level indicators of social outcomes influence positively on the meso-level of NPO outcomes.

## Methodology

### Data Collection and Sample Description

In order to confirm the established conceptual model, we have used NPOs as the unit of analysis. We think that it is more likely that NPOs, to the extent that their ultimate indicator of results is the fulfillment of a certain social mission (Vázquez et al., 2002; McDonald, 2007), have greater knowledge than companies regarding to what extent the co-creation between both entities can contribute to: (1) the satisfaction of the demands, needs, and expectations of the beneficiaries and users of the results of the collaboration (activities, programs, services, etc.), and (2) its impact on the community and on the sectoral conditions (e.g., environmental, social, education, health, economic, etc.) of the society in which the collaboration takes place.

A census was carried out including Spanish NPOs that potentially collaborate or have collaborated with firms in the development of projects, programs, etc. This census was necessary in the absence of an analogous one in public records. For its preparation, around 20 secondary information sources were used and classified as follows: (1) directories of socially innovative organizations, (2) specific forums for SI, (3) crowdfunding solidarity platforms, (4) awards for innovation, entrepreneurship, or social transformation, (5) studies on Spanish NPOs, and (6) networks or projects of social entrepreneurship. The result of this process was a census of 358 NPOs.

An online survey was carried out among the NPOs comprising the census. After prior telephone contact requesting collaboration in the study, 358 NPOs received access to an online structured questionnaire *via* e-mail. The recipient of the questionnaire was the person in charge of the daily management and ordinary decision-making of the organization. The questionnaire was structured as follows: after a first question related to the degree of development of the collaborative culture within the respective NPO, a dichotomous question was included, as a filter, to be able to identify those NPOs that collaborate or would have collaborated with private companies to carry out the referred projects. If so, a series of sequential questions were posed, aimed, among other questions, at evaluating the degree of business-NPO co-creation in a specific collaboration (the main or most representative), as well as its consequences at the micro, meso and macro levels of analysis. Data collection took place between January 2018 and May 2018. The final sample included 205 NPOs. Stratified sampling was used. Table 1 shows the description of the sample (sample error of ± 4.5%).

**Table 1:** Sample description.

**Description**		**Census (*N* = 358)**	**Sample (*n* = 205)**
Year of constitution	Until 1978	5.8%	5.5%
	1979–1994	23.0	25.0
	1995–2002	26.5	30.5
	After 2003	44.7	39.0
Legal form	Associations	45.0	45.9
	Foundations	55.0	54.1
Founders	Natural persons	72.2	74.7
	Legal persons	40.4	40.2
	Public legal persons	7.6	5.2
	Private legal persons	38.6	39.2
	Private legal persons: business	12.9	11.3
	Private legal persons: other NPOs	21.9	22.7
	Private legal persons: others	10.8	10.8
Beneficiaries	Legal persons	23.5	23.9
	Natural persons	95.3	93.2
	Natural persons: society	38.3	33.2
	Natural persons: specific groups	79.1	79.5
International classification of non-profit organizations (ICNPO)	Culture/Recreation	15.9	14.1
	Education/Research	53.4	48.8
	Social services	59.5	60.0
	Health	19.3	19.5
	Environment	14.0	9.3
	Development/Housing	21.5	22.0
	Law, advocacy, and polities	12.6	11.7
	International	26.8	22.4
	Religion	1.4	0.5
	Business, professional associations, and unions	7.3	6.8
Scope	Regional	41.8	43.9
	National	33.1	35.7
	International	25.1	20.4
Size	Micro-sized (revenue < €30,000)	6.4	6.5
	Small-sized (€30,000–500,000)	35.3	34.1
	Medium-sized (€500,000–2,400,000)	30.8	28.6
	Large/Mega-sized (revenue >€2,400,000)	27.4	30.8

We employed various techniques to assess the possible existence of non-response bias. First, we compared sample descriptors of the 205 NPOs with descriptors of the population. In this case, it has been observed that there are no statistically significant differences between them. Second, we compared two groups of respondents. The first group, consisting of early respondents who returned their response after a single previous contact, was composed of 139 NPOs. The second group, whose data was obtained later as an extra effort of non-response follow-up, involved 66 NPOs. The estimation of a two sample (independent) *t*-test reveals that there were no statistically significant differences between the two groups of respondents.

### Measures

To develop a valid and reliable measurement scale of NPO-business value co-creation, we followed the study of Churchill (1979) and Netemeyer et al. (2003). First, we generated a tentative scale of NPO-business value co-creation from the four critical dimensions of “co-production” identified by Bharti et al. (2015). We named them as follows: (1) participation, (2) reciprocity, (3) learning, and (4) engagement. The result was the first relationship of 31 items. The items used to measure participation and reciprocity have been obtained from Bharti et al. (2015), the items corresponding to the learning dimension have been obtained from Sanzo et al. (2012), and the items of the engagement dimension from Vivek et al. (2014). In all the cases, we have employed seven-point item scales.

Furthermore, a Delphi Analysis was carried out with the collaboration of nine researchers and/or managers in the field of corporate social responsibility, SI, value co-creation, non-profit marketing, or NPO management. As a result of this analysis, we incorporated several modifications to the initial set of 31 items. Specifically, some items were included, items with a similar meaning were grouped, and/or other items difficult to understand or needed to be adapted to the research context were reformulated. At the end of the process, the scale used in the questionnaire was comprised of 32 items.

We also used seven-point item scales (see Appendix) to analyze the consequences of adopting the NPO-business value co-creation strategy. All of them were reflective scales. First, the two-item scale of micro level consequences, that the development of an NPO co-creation strategy has on its beneficiaries or more direct users, was adopted from Modi and Mishra (2010) and Sanzo et al. (2015). We measured the consequences of the NPO-business value co-creation has on the NPO organizational performance by means of 13 items, based on the scales used by Zappalà and Lyons (2009) and Modi and Mishra (2010), and Sanzo et al. (2015). For its part, we measured the meso-level benefits of adopting this value co-creation strategy for the collaborating company (as perceived by the NPO) with a scale consisting often items derived from Bharti et al. (2015), Modi and Mishra (2010), and Sanzo et al. (2015). The seven-item scale was adopted from Modi and Mishra (2010) and Sanzo et al. (2015) in order to measure the consequences the development of this strategy has at the macro level (society).

## Results

### Measurement Models

We first analyzed the business-NPO value co-creation scale with confirmatory factor analysis (CFA), using STATA version 13.1. The estimation method employed was the maximum likelihood. To obtain the best possible fit, three alternative models were estimated sequentially, by eliminating the items that caused a lack of adjustment in the initial model. The elimination of these items was done considering three criteria (Jöreskog and Sörbom, 1993): (1) the elimination of those indicators that have a weak convergence condition with their corresponding latent variable (a Student's *t*-distribution greater than 2.58 is required for *p* = 0.01; none of the items have been eliminated as a consequence of this criterion); (2) the elimination of those variables with standardized coefficients of less than 0.5, considered as a strong convergence criterion; and (3) the elimination of those indicators that have a linear *R*^2^ ratio lower than 0.3. The Appendix shows the means and SDs of the 19 items included in the final business-NPO co-creation scale.

The goodness-of-fit indices of the final scale of business-NPO value co-creation are appropriate [Chi-Square = 284.321 (*p* = 0.000); Chi-Square reasons/degrees of freedom = 1.947; comparative fit index (CFI) = 0.938; root mean square residual (RMSR) = 0.054; root mean square of approximation (RMSEA) = 0.077]. Tables 2, 3 reveal the existence of reliability, convergent validity, and discriminant validity regarding the final four dimensions of value co-creation.

**Table 2:** Confirmatory factor analysis (CFA) of the business-NPO value co-creation measurement model.

**Factor**	**Item**	**Factor loadings**	**Composite reliability coefficient**	**AVE**
Participation (P)	P_1	0.818^***^	0.912	0.777
	P_2	0.958^***^		
	P_3	0.862^***^		
Reciprocity (RE)	RE_1	0.680^***^	0.857	0.547
	RE_2	0.712^***^		
	RE_3	0.759^***^		
	RE_4	0.803^***^		
	RE_5	0.737^***^		
Learning (LEARN)	LEARN_2	0.864^***^	0.879	0.647
	LEARN_3	0.700^***^		
	LEARN_4	0.888^***^		
	LEARN_5	0.749^***^		
Engagement (ENG)	ENG_1	0.558^***^	0.924	0.642
	ENG_4	0.800^***^		
	ENG_6	0.818^***^		
	ENG_8	0.880^***^		
	ENG_10	0.924^***^		
	ENG_11	0.922^***^		
	ENG_13	0.626^***^		

*** *p < 0.01*.

**Table 3:** Discriminant validity of the value co-creation scale.

	**P**	**RE**	**LEARN**	**ENG**
**P**	**0.777**			
**RE**	0.500^***^	**0.547**		
**LEARN**	0.326^***^	0.479^***^	**0.647**	
**ENG**	0.331^***^	0.534^***^	0.130^***^	**0.642**

*The values on the diagonal are the AVE coefficients of each of the four constructs. The values off the diagonals are the square of the correlations between each pair of constructs*.

*** *p < 0.01. The bold indicates AVE coefficients of each of the four constructs*.

A CFA was employed to test the reliability, convergent validity, and discriminant validity of the consequences of business-NPO value co-creation, using the estimation method of the maximum likelihood.

Table 4 shows that goodness-of-fit indices are appropriate, except for CFI, which is close to a good fit. However, this goodness-of-fit is achieved to the extent of considering two latent dimensions, when explaining the perception that the NPO has about the company performance with which it collaborates. On the one hand, the perception about the satisfaction of the expectations of the company with the collaboration experience. On the other hand, the perception about the impact on the competitive positioning of the company through an image and a range of products and services more valuable to its customers. Thus, the model constructs are reliable, since all the factors have a composite reliability coefficient higher than the reference value of 0.7. Likewise, the analysis of the average variance extracted (AVE) shows values greater than 0.5 for all the constructs. There is a statistical significance between each indicator and its factor, and the standardized coefficients of all the factors are greater than the minimum recommended value of 0.5, so the convergent validity criterion is met. In addition, discriminant validity exists, since the square of the correlations between each of the factors is less than the AVE of the constructs involved (Table 5).

**Table 4:** Confirmatory Factor Analysis (CFA) of the measuring model of the consequences of the business-NPO value co-creation.

**Factor**	**Item**	**Factor loadings**	**Composite reliability coefficient**	**AVE**
Value co-creation (COCR)	P	0.784^***^	0.878	0.647
	RE	0.963^***^		
	LEARN	0.674^***^		
	ENG	0.768^***^		
Consequences of micro level (B1)	B1_1	0.742^***^	0.804	0.674
	B1_2	0.893^***^		
Consequences of meso level-NPO (B2)	B2_4	0.754^***^	0.797	0.567
	B2_5	0.735^***^		
	B2_8	0.770^***^		
Consequences of meso level—business value (B3)	B3_1	0.738^***^	0.805	0.583
	B3_2	0.883^***^		
	B3_3	0.651^***^		
Consequences of meso level—business satisfaction (B4)	B4_1	0.845^***^	0.935	0.784
	B4_2	0.907^***^		
	B4_3	0.910^***^		
	B4_4	0.880^***^		
Consequences of macro level (B5)	B5_5	0.697^***^	0.816	0.602
	B5_6	0.919^***^		
	B5_7	0.689^***^		

*Chi-Square = 935.658 (p = 0.000); Chi-Square reason/degrees of freedom = 1.838; CFI = 0.884; RMSR = 0.079; RMSEA = 0.076*.

*** *p < 0.01; CFI, comparative fit index; RMSR, root mean square residual; RMSEA, root mean square of approximation*.

**Table 5:** Discriminating validity of the consequences of the business-NPO value co-creation.

	**COCR**	**B1**	**B2**	**B3**	**B4**	**B5**
**COCR**	**0.647**					
**B1**	0.142^***^	**0.674**				
**B2**	0.430^***^	0.460^***^	**0.567**			
**B3**	0.321^***^	0.198^***^	0.529^***^	**0.583**		
**B4**	0.336^***^	0.339^***^	0.312^***^	0.250^***^	**0.784**	
**B5**	0.181^***^	0.124^***^	0.308^***^	0.254^***^	0.023^*^	**0.602**

*The values on the diagonal are the average variance extracted (AVE) coefficients of each of the five constructs. The values off the diagonal are the squares of the correlations between each pair of constructs*.

*** *p < 0.01*;

* *p < 0.1. The bold indicates AVE coefficients of each of the six constructs*.

### Structural Model of the Consequences of NPO-Business Value Co-creation

**Figure 2:**
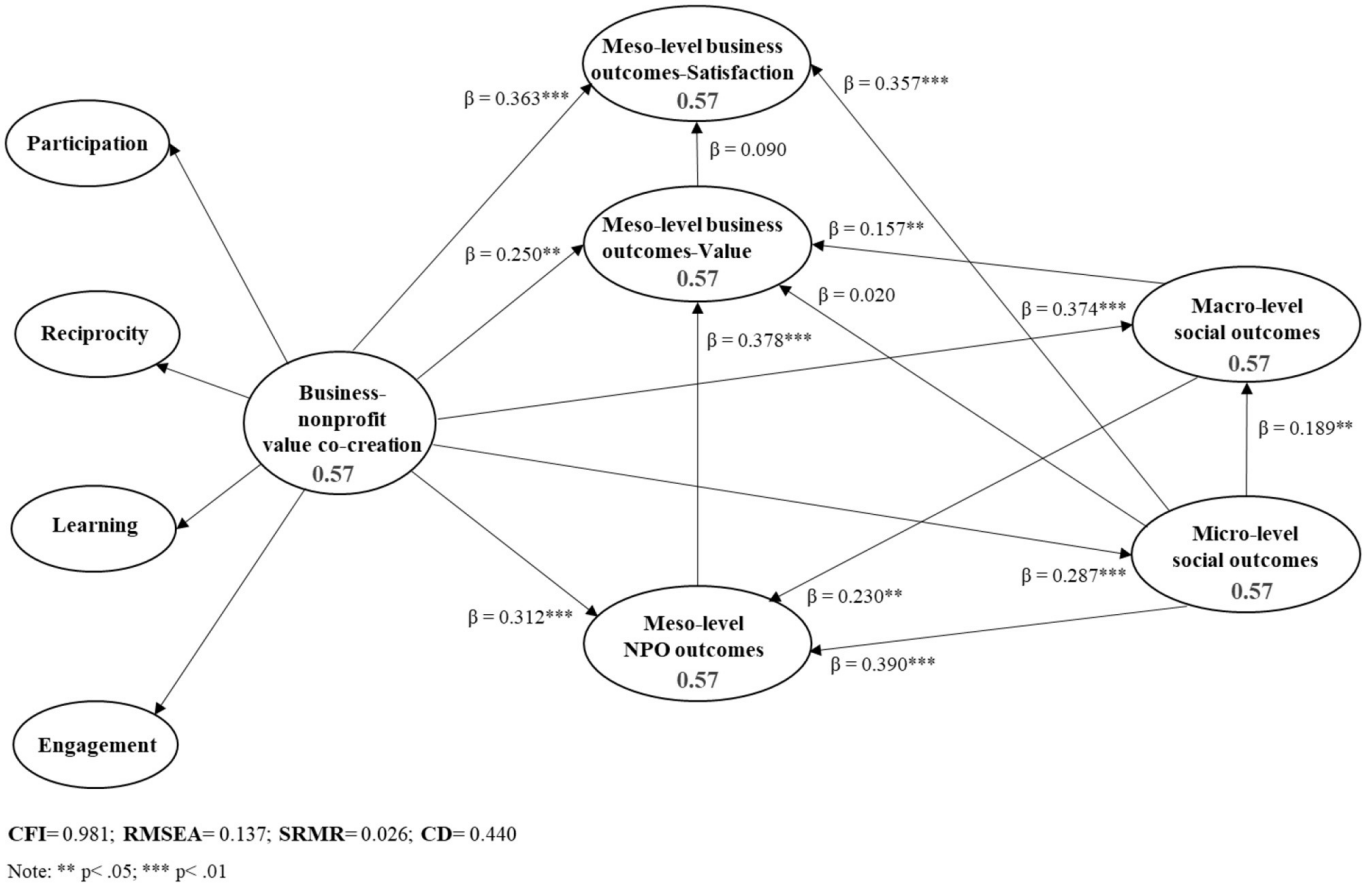
Causal model of the consequences of the business–NPO value co-creation.

We tested the research hypotheses with structural equations modeling (SEM) analysis, using STATA 13.1 (Figure 2). The estimation method employed was the maximum likelihood. Goodness-of-fit indices of the model are appropriate, except for RMSEA, which is close to a good fit.

First, business-NPO value co-creation is positively associated with improved performance and satisfaction indicators of the company, so H1.a and H1.b are accepted (*p* < 0.05 and *p* < 0.01, respectively). Likewise, business-NPO value co-creation is positively associated with improved performance indicators of the NPO, so H1.c (*p* < 0.01) is supported.

Second, in the case of the company, the NPO perceives that the development of a co-creation strategy increases the satisfaction of the company with the collaboration directly, but not indirectly through the effect that this strategy has on the improvement of its competitive position in the market. In addition, the results show that indeed NPO performance indicators are positively associated with improved company performance indicators, so H2.b is accepted (*p* < 0.01).

Third, business-NPO value co-creation is positively associated with the micro- and macro-level indicators of social outcomes, so H3.a (*p* < 0.01) and H3.b (*p* < 0.01) are supported. It is confirmed as the positive impact of co-creation on the macro-level indicators of social outcomes are indirectly stimulated by the effect of this strategy on the satisfaction of the demands, needs, and expectations of the beneficiaries and users of the results (activities, programs, services, etc.) of the collaboration (micro level indicators). Therefore, the H3.c (*p* < 0.05) can be considered verified.

Finally, the micro-level indicators of social outcomes have no significant effects on the improvement of the competitive position of the company. However, results show that the micro-level indicators are positively associated with the perception of the NPO regarding the increase in the satisfaction of the company with the collaboration, as well as with the improvement of the NPO performance indicators, in line with H4.b (*p* < 0.01) and with H4.c (*p* < 0.01). Furthermore, the macro-level indicators of social outcomes are positively associated with the improvement of the performance indicators of both the company and the NPO, so H4.d (*p* < 0.05) and H4.e (*p* < 0.05) are accepted.

## Discussion

Social innovation is a complex phenomenon, analyzed from different lines of research, in which very diverse organizations are involved. It includes a myriad of activities that share four basic characteristics: (1) they are innovations aimed at solving serious current social problems, which are reflected in the SDGs; (2) in their development, they incorporate collaborative processes with the participation of the main stakeholders involved, (3) with a long-term sustainable use of resources, and (4) generate changes in social behaviors and practices.

Social innovation is not an exclusive activity of NPOs. The evolution that has taken place over the years in the understanding of corporate social responsibility has meant that SI is also seen as a key strategy by an increasing number of companies from a wide variety of sectors. Given that an essential feature of SI is its collaborative nature and the empowerment of stakeholders, especially those from the most vulnerable groups, a factor that could promote these activities in companies would be the development of partnerships with NPOs, where value co-creation activities were involved.

With this research, we have explored the effect of business-NPO value co-creation not only on their respective performance, but also on their impact on 2030 United Nations SDG, to the extent that this cross-sectoral alliance can contribute to strengthening the implementation and revitalization of the global partnership for sustainable development (Seth, 2015). In fact, according to (von Schnurbein, 2020, p. 1) unlike the Millenium Development Goals, characterized by **“**the division of the world into donors and recipients of assistance was accentuated as one of the limitations to success, although a global partnership was proclaimed as one of eight goals” (Wysokinska, 2017), the SDGs focus on value co-creation processes for their effective fulfillment.

To this end, NPOs have been used as the unit of analysis, as entities whose key focus of action is increasingly improving the unmet social needs through socially innovative solutions (Anheier et al., 2019) that will have consequences on the community and on the sectoral conditions of the society. Several relevant conclusions have been drawn from the results obtained.

Firstly, it is perceived that companies can improve not from their competitive position in the market through value co-creation with NPOs, an expected consequence according to the specialized literature on value co-creation (Bharti et al., 2015), but from sustainable development parameters that comply with the SDGs. The company can see how its organizational performance increases when the NPO improves its results as a consequence of co-creation, confirming the “transferred resource value” generated in the relationship; in other words, the benefits obtained by a partner from the resources obtained by the other party in the co-creation process (Austin and Seitanidi, 2012a). Specifically, the company can take advantage of the NPO experience and the knowledge of its environment to improve its own management processes, which could have an impact on commercial offers of greater social value for its customers.

Secondly, the development of this business-NPO value co-creation strategy has direct consequences on social welfare, reflected in the satisfaction of the most pressing needs, desires, and expectations of the recipients or users of the initiatives in which the co-creation is embodied, as in the positive impact generated in the community, and social environment in which they grow, by accessing better social, environmental, educational, health, and economic conditions. Therefore, the importance of co-creation in partnerships between companies and the non-profit sector for the fulfillment of the SDGs is highlighted, as recently stated by von Schnurbein (2020), contributing to “enhance the global partnership for sustainable development, complemented by multi-stakeholder partnerships that mobilize and share knowledge, expertise, technology, and financial resources, to support the achievement of the sustainable development goals in all countries, in particular developing countries” (Assembly, 2017, p. 21).

However, despite the global multi-stakeholder partnerships is promoted within the framework of the SDGs, studies have shown that they are challenged by unbalanced participation (particularly, the limited participation of stakeholders from sub-Saharan Africa), which leads to “suboptimal outcomes of partnerships” (von Schnurbein, 2020, p. 12). In line with this research, a solution could be the adoption of value co-creation processes, such as SI mechanisms (Voorberg et al., 2013), that put the resolution of current and complex social problems in the focus of collaboration through the active and effective participation of the partners with the beneficiaries (Bharti et al., 2015), which would lead to the optimal achievement of the consequent outcomes of the process. And, taking into account the long-term engagement generated by the co-created activities, new collaboration contracts could be established in the future by current and potential partners.

Then, the inclusive participation of the recipients of the co-creation is crucial in order to achieve a greater and more effective positive social impact in the fulfillment of the SDGs. In this sense, when both organizations co-create, the satisfaction of the beneficiaries with the projects in which they have participated, their empowerment, and greater access to resources, leads to an optimal opportunity for the community to achieve sustainable economic growth. Therefore, the interrelation between both the levels of results, micro (beneficiaries) and macro (social impact), has a multiplier effect for the achievement of the SDGs.

Thirdly, we can see how the improvement of the quality of life of benefits and users, and the impact achieved in the social environment as a result of collaboration contribute to the organizational performance of companies and NPOs from parameters of SI (Howaldt and Schwarz, 2010). On the one hand, company managers should appreciate the fact that co-creation with NPOs will contribute to strengthening the image and competitive position of the entity in the market. On the other hand, NPO managers will perceive that through this strategy, they will be able to increase the visibility, reputation, and/or legitimacy of the organization in the eyes of society, or see an increase in the number of entities (other companies, NPOs, etc.) that wish to collaborate. This could mean an increase in their benefits; therefore, a more effective fulfillment of their mission in the different social environments is concurrent with the SDGs.

Fourthly, the evaluated conceptual model shows us that the benefits the users obtain from the activities and services provided cannot be linked to the perception of the NPO —that the company achieves better performance with the collaboration. A possible explanation could be found in the fact that the NPO considers its organizational mission strongly rooted in the positive results achieved by the beneficiaries of the projects and programs carried out, and that such compliance is not so closely related to the returns obtained by the company. Furthermore, the improvement of the performance of the company by co-creating with the NPO cannot be related to the perception of the latter regarding the satisfaction of the company in the collaboration, which shows that the company is truly committed to achieving social improvement and attaining the SDGs, beyond its own individual outcome. In any case, value co-creation, considered a key strategy within the corporate social responsibility policies and practices of companies (Vargo and Lusch, 2016), is an extremely valuable approach to contribute to the achievement of the SDGs from a business perspective based on the principles of SI.

## Conclusion

With this research we have explored the effects of business-NPO co-creation projects on micro- (individuals), meso- (organizations), and macro(society)-level results, as a means to improve the business performance and, simultaneously, the community welfare and the achievement of SDGs. The contribution is two-fold. First, the study contributes to Corporate Social Responsibility literature by providing insights about the consequences of a “shared value” approach (Porter and Kramer, 2011). Second, we assessed these consequences from the NPO viewpoint, an approach that can offer a more accurate perspective of the social impact resulting from co-creating with businesses, as an “inescapable and powerful vehicle for implementing CSR and for achieving social and economic missions” (Austin and Seitanidi, 2012a, p. 728). Therefore, companies and NPOs that want to move toward solving social and environmental challenges by meeting the SDGs must take into account, among the strategies used, co-creation processes, as a type of SI, where effective participation, established long-term links, consequent learning of the relationship, and reciprocity in the actions of the partners allow to successfully achieve the established goals.

The main limitation is that the study adopted a cross-sectional character. Another one is that the investigation has been based on one of the parts of the relationship, and there may be possible dissonances between the perceptions of the NPOs and those of the collaborating companies regarding the type of relationship that both maintain. Research could be improved by incorporating other possible consequences of NPO–business value co-creation (e.g., to analyze aspects related to the effectiveness and the efficiency of the programs developed and to the effectiveness in the management of the NPOs). Likewise, future research could evaluate, with the aim of increasing the knowledge of the consequences of adopting a value co-creation, strategies, and differences between groups that allow to observing the existing diversities among NPOs that, in addition to collaborating with companies, co-create with other NPOs or with the government.

## Data Availability Statement

The raw data supporting the conclusions of this article will be made available by the authors, without undue reservation.

## Author Contributions

YD-P organized the databas and performed the statistical analysis. YD-P, LÁ-G, and MS-P contributed to conception and design of the study and wrote sections of the manuscript. All authors contributed to manuscript revision, read, and approved the submitted versión.

## Conflict of Interest

The authors declare that the research was conducted in the absence of any commercial or financial relationships that could be construed as a potential conflict of interest.

## Publisher's Note

All claims expressed in this article are solely those of the authors and do not necessarily represent those of their affiliated organizations, or those of the publisher, the editors and the reviewers. Any product that may be evaluated in this article, or claim that may be made by its manufacturer, is not guaranteed or endorsed by the publisher.

## Appendix

**Table:**

**Business-NPO value co-creation (COCR)**		
**Participation (P):** The extent to which the company carries out the following activities.		**Mean (S.D.)**
P_1	The company shares with us relevant information that can be used in the different stages of the collaboration processes.	6.28 (1.059)
P_2	The company provides suggestions for these collaboration processes.	6.21 (1.033)
P_3	The company participates in decision-making regarding one or more stages of the collaboration.	5.93 (1.307)
**Reciprocity (RE):** The extent to which you agree with the issues listed below.		**Mean (S.D.)**
RE_1	Even if the relationship's costs and benefits are not equivalent at a certain moment of time, they are balanced in the long term.	4.59 (1.818)
RE_2	We believe that the relationship is characterized by the fact that each partner learns from the other.	4.91 (1.667)
RE_3	Both organizations jointly review past experiences to learn from successes and mistakes.	4.49 (1.824)
RE_4	We both like to reconsider frequently how to do things and we are willing to change in order to adapt to new circumstances.	4.65 (1.797)
RE_5	Both organizations share the same goal with collaboration, to which we are committed.	5.21 (1.676)
**Learning (LEARN):** The extent to which you consider that the relationship has the following characteristics.		**Mean (S.D.)**
LEARN_2	The company gets information from us that can be helpful in its own activities or processes.	4.89 (1.890)
LEARN_3	We believe that such information is spread, shared, and/or applied within its organization.	4.53 (1.738)
LEARN_4	We believe that this information allows the company to be more efficient and/or to better perform its activities.	4.33 (1.881)
LEARN_5	We believe that the company introduces changes in its management or in the way it operates, as a result of collaborating with us.	3.65 (1.913)
**Engagement (ENG):** The extent to which you think the company shows the following characteristics when collaborating with your entity.		**Mean (S.D.)**
ENG_1	Company executives prove to be very committed to collaboration.	5.29 (1.490)
ENG_4	Business partners who collaborate with us show a lot of interest in and attention to the project, program, etc., particularly where we collaborate.	5.62 (1.484)
ENG_6	Business partners who collaborate with us take the necessary time to carry out the collaboration objectives.	5.14 (1.556)
ENG_8	Such interlocutors prove to be personally involved in the collaboration.	5.04(1.684)
ENG_10	Business partners seem to enjoy the collaboration a lot.	5.27 (1.599)
ENG_11	Business partners who collaborate with us enjoy the teamwork.	5.08 (1.653)
ENG_13	The relationship developed between our staff and the business partners extends beyond the professional relationship, creating personal ties.	3.85 (2.126)

*As a consequence of the validation of the scale, some items have been eliminated*.

**Table:**

**Consequences of the micro level (B1):** The extent to which the collaboration with the company had an		**Mean (S.D.)**
impact on the following performance indicators.		
B1_1	Tangible results obtained by the beneficiaries or users of the entity as a result of the collaboration (for example, having accessed more social benefits, programs, etc.).	5,41 (1,603)
B1_2	Satisfaction of the demands, needs, and expectations of the beneficiaries or users.	5,47 (1,421)
**Consequences of the meso level-NPO (B2):** The extent to which the collaboration with the company had an		**Mean(S.D.)**
impact on the following performance indicators.		
B2_1	Increase in the income of the entity.	4,78 (1,970)
B2_2	Increased economic efficiency in management (the result of the collaboration exceeds the resources used for its development).	4,34 (1,838)
B2_3	Increase in the number of employees or volunteers who wish to collaborate with the entity.	3,90 (1,857)
B2_4	Increase in the number of organizations (other companies, non-profit organizations, etc.) that want to collaborate with the entity.	4,48 (1,825)
B2_5	Improving staff satisfaction (employees/volunteers).	4,81 (1,725)
B2_6	Increased productivity or staff efficiency.	4,23 (1,743)
B2_7	Introduction of “good practices” (ethical or transparency codes.) in the entity.	4,40 (1,918)
B2_8	Increased visibility, reputation and/or legitimacy of the entity with the society.	5,49 (1,403)
B2_9	Number of final and direct beneficiaries of the projects, programs, and social benefits of the entity.	5,26 (1,574)
B2_10	Use of impact or scope evaluation systems (audits, logical framework, social return on investment, or SROI.) of said projects, programs, and social benefits.	4,15 (1,996)
**Consequences of the meso level-Bbusiness Value (B3):** The extent to which the collaboration with the company had an		**Mean(S.D.)**
impact on the following performance indicators.		
B3_1	Improving the image of the collaborating company.	5,57 (1,363)
B3_2	Improvement of the competitive position of the collaborating company.	4,72 (1,766)
B3_3	Development by the company to provide more valuable products and/or services for its clients.	4,05 (1,858)
**Consequences of the meso level-business Satisfaction (B4):** The extent to which the collaboration with the company had an		**Mean(S.D.)**
impact on the following performance indicators.		
B4_1	Satisfaction of the expectation of the company on the use of the resources provided with the collaboration.	5,51 (1,210)
B4_2	Satisfaction of the expectation of the company regarding the development of the project in which it collaborates.	5,67 (1,173)
B4_3	Satisfaction of the expectation of the company regarding the impact of the aforementioned project or program.	5,55 (1,211)
B4_4	Overall satisfaction of the company with the collaboration experience developed.	5,76 (1,093)
**Consequences of the macro level (B5):** The extent to which the collaboration with the company had an		**Mean(S.D.)**
impact on the following performance indicators.		
B5_1	Increased awareness of the social cause to which the collaboration is linked.	5,20 (1,635)
B5_2	Increased citizen support for the social cause.	4,20 (1,806)
B5_3	Increased influence capacity of the citizens.	4,05 (1,825)
B5_4	Increased influence of social cause on the political agenda.	3,41 (1,838)
B5_5	Changes in legislation or reforms in favor of the social cause.	2,47 (1,623)
B5_6	Creating opportunities for sustainable economic growth in the community.	3,52 (1,852)
B5_7	Improvement of the sectoral conditions (for example, environmental, social, educational, health, economic, etc.) of the society in which the collaboration takes place.	4,12 (1,953)
